# Structural Recognition and Maternal Mental Health in Muslim-Majority States

**DOI:** 10.1192/j.eurpsy.2026.11933

**Published:** 2026-07-31

**Authors:** A. A. Rehman, A. Gasparova, B. R. Carr

**Affiliations:** University of Florida, Gainesville, FL, United States

## Abstract

**Introduction:**

Maternal health has become a global benchmark of national progress, yet in many Muslim majority countries, particularly across North and East Africa and South Asia, the focus remains overwhelmingly physical. Mortality is measured, while psychological suffering is left unaddressed. Even when postpartum depression, anxiety, or trauma are documented, they are often treated as secondary—stigmatized, poorly defined, and institutionally neglected. This paper explores that invisibility through a comparative analysis of Pakistan, Egypt, Sudan, Morocco, Somalia, and Afghanistan. These countries differ in language, political history, and health infrastructure, yet they share structural conditions that suppress recognition of maternal psychological distress. The result is not merely a service gap, but a deeper ethical failure that renders maternal mental health systematically unseen.

**Objectives:**

This study examines how maternal psychological suffering is either acknowledged or excluded within six Muslim majority contexts. We explore the institutional, clinical, and cultural frameworks that contribute to this neglect, and argue that maternal mental health is not only underserved but actively displaced and treated as too intimate, too ambiguous, or too entangled with culture to be addressed as a public responsibility.

**Methods:**

A comparative interpretive review was conducted across peer-reviewed literature published since 2016, focusing on studies using validated tools and examining social or structural determinants of maternal mental health. Four thematic dimensions guided analysis: recognition in policy and training, diagnostic prioritization of physical over psychological risk, the buffering role of informal care networks, and patterns of disclosure shaped by stigma, violence, and silence.

**Results:**

Across all six countries, psychological conditions remain marginalized. Symptoms are often reinterpreted through moral or spiritual language, while institutional responses are minimal or nonexistent. Informal networks may offer emotional relief but also serve as substitutes for formal care, shifting responsibility away from the state. The pattern is consistent: maternal mental illness is measurable and visible in research but remains absent from health systems and clinical routines.

**Conclusions:**

Maternal mental health in Muslim majority contexts is not simply neglected, it is systematically denied recognition. This denial is not incidental but embedded in how health priorities are constructed, measured, and resourced. Recognition must go beyond symbolic gestures and be woven into clinical infrastructure, public health policy, and ethical frameworks. Maternal psychological suffering must be seen as a core concern of justice and collective responsibility. Until this occurs, maternal health efforts will remain incomplete, and the interior lives of mothers will continue to be rendered invisible.

**Disclosure of Interest:**

None Declared

